# Deep Fat Saving Elevation of the Superficial Circumflex Iliac Artery Perforator Flap

**DOI:** 10.3390/medicina58050670

**Published:** 2022-05-18

**Authors:** Yuma Fuse, Hidehiko Yoshimatsu, Ryo Karakawa, Tomoyuki Yano

**Affiliations:** Department of Plastic and Reconstructive Surgery, Cancer Institute Hospital, Japanese Foundation for Cancer Research, Tokyo 135-8550, Japan; hidehiko.yoshimatsu@gmail.com (H.Y.); ryo.kyara@gmail.com (R.K.); yanoaprs@icloud.com (T.Y.)

**Keywords:** superficial circumflex iliac artery perforator flap, microsurgery, soft tissue tumor

## Abstract

*Background and Objectives*: Prolonged drain stay and lymphorrhea are often problems at the donor site of the superficial circumflex iliac artery perforator (SCIP) flap. This study aimed to introduce a novel technique of the SCIP flap elevation: Deep Fat Saving (DFS) technique. *Materials and Methods*: Thirty-two patients who underwent the SCIP flap transfer were divided based on the flap-elevated layer: above the deep fascia or the Camper fascia saving the deep fat. The duration of drain stay and the rates of flap survival and donor-site complications were compared between the groups. The inverse probability weighting (IPW) method was conducted to balance confounders. *Results*: By IPW, two balanced pseudo-populations were created: DFS = 33.9 and Conventional = 31.3. There were no significant differences in the rate of flap survival (DFS: 100% verses Conventional: 95.8%, *p* = 0.32) and donor site complications (DFS: 2.4% versus Conventional: 1.3%, *p* = 0.68, respectively). The duration of drain stay was shorter in the DFS group (weighted median: 6 versus 8 days; weighted difference: −1.6 days (95% confidence interval: −2.8 to −0.4), *p* = 0.01). *Conclusions*: An SCIP flap can be reliably harvested using the Deep Fat Saving technique.

## 1. Introduction

The use of the superficial circumflex iliac artery (SCIA) perforator (SCIP) flap has become popular as its anatomy was well described [1,2,3,4,5]. The elevation technique is safe, and the scar can be concealed. In addition, the donor site morbidity is low [5]. The SCIP flap has been mainly used for extremity reconstruction. Recent advances have expanded its use ranging from head-and-neck to breast to toe reconstruction [6,7,8]. Each of the SCIA branches is used as the pedicle; the skin flap is based on the superficial branch, and the sartorius muscle or the iliac bone flap is harvested based on the deep branch. By combining these branches, a chimeric flap can also be elevated [4].

The conventional SCIP flap is harvested on the deep fascia, including the whole fat layers [9]. Amongst donor-site morbidities of the SCIP flap are prolonged drain stay and lymphorrhea that develop due to damaging the lymphatic vessels in the deep fat. One study reported that one out of 210 patients developed lymphorrhea after the SCIP flap elevation [10]. Recently, in pursuit of ideal resources, a thinner flap has been used for face or extremity reconstruction in which a skin flap is elevated above the superficial fascia [10,11,12,13,14,15]. Although this is less invasive to the donor site with the deep fat remained, the technique is challenging.

As we routinely use the SCIP flap, we have noticed a distinct fascia between the superficial and the deep fascia. We hypothesized that flap elevation above this fascia enables to save the deepest fat in a safer way as a modification of the conventional procedure, contributing to minimizing the invasiveness and enhancing the reliability simultaneously.

In this study, we introduce our modification of SCIP flap elevation saving a thin layer of the deeper fat tissue. We also compared the reconstructive result and donor site morbidity.

## 2. Materials and Methods

After the approval of the Institutional Review Board at Cancer Institute Hospital (2021-GB-101), all patients who had undergone the SCIP flap reconstruction with sarcoma excision by senior surgeons (YF, RK, and HY) from January 2020 through December 2021 were identified retrospectively. We excluded patients who underwent the SCIP flap transplant based on the deep branch of the SCIA, those who had non-spindle shaped SCIP flap transplant or those who had previous operations in the abdomen. The collected data included patient demographics, the location of the defect, the flap size, the elevation layer, the total operative time, the survival of flap, the duration of drain stay, the development of donor-site complications such as seroma and lymphocyst during the postoperative one month, and the necessity of revision to correct the bulkiness of the flap.

### 2.1. Deep Fat Saving Technique

We elevated the SCIP flap in either the supine or the lateral decubitus position under general anesthesia. The superficial branch of the SCIA was identified using a high-resolution ultrasound system (Vevo MD ultrasound device, Fujifilm Visual Sonics, Amsterdam, the Netherlands) [16]. The flap size was decided in accordance with the defect dimension, and a spindle-shaped skin paddle was designed along the superficial branch. Based on careful consideration of the defect thickness, an SCIP flap was elevated conventionally above the Scarpa fascia or using the “deep fat saving technique”.

**Figure 1:**
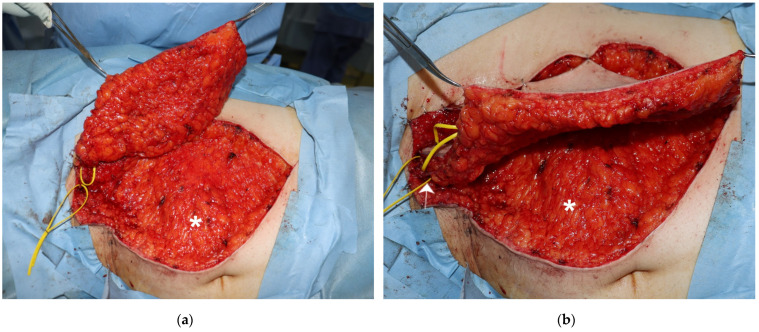
Deep Fat Saving technique. (**a**) The deep fat (*) was saved in an SCIP flap elevation; (**b**) a relatively thick skin flap was harvested based on the superficial branch of the SCIA (white arrow).

An SCIP flap was elevated in a proximal-to-distal fashion [9]. A 3–4 cm skin incision was made along the pedicle marking. The pedicle was identified in the fat tissue and dissected proximally for the required length. The skin was then cut along the flap design. The fat tissue was dissected down. During the dissection, the superficial fascia was exposed and incised. Between the superficial and the Scarpa fasciae, another white fascia that is referred to as the Camper fascia was identified [17]. The skin flap was elevated above this fascia, and the deeper fat was saved (Figure 1). After the elevation was completed, the flap perfusion was confirmed on indocyanine green angiography [18]. Recipient vessels were prepared within or in the adjacent of the defect after tumor excision. The free SCIP flap was transplanted, and microsurgical anastomosis was established.

The donor wound was closed directly over one suction drain of 15 Fr (BLAKE drain, Ethicon, Bridgewater, NJ, USA). The drain was maintained for at least two days and removed if discharge was less than 20 mL per day.

### 2.2. Statistical Analysis

Numeric variables were described using median and interquartile range (IQR), and categorical variables were shown using number and proportion. We compared the frequency of the postoperative morbidities (total or partial necrosis of the SCIP flap and donor site complications) and the duration of drain stay between the conventional and the DFS groups. The inverse probability weighting (IPW) method was used to minimize the potential imbalances between the groups [19]. The propensity score estimating the probability of receiving the Deep Fat Saving technique was calculated using a logistic regression model with the following valuables: age, sex, body mass index (BMI), smoking history, preexisting medical conditions, the dimension of the flap, and the location of the defect. Age was categorized into two groups: <65 and ≥65 years. BMI was grouped into two: <25 and ≥25 kg/m2. These predictor factors were chosen based on consensus amongst the investigators. The thresholds categorizing the patients were decided in accordance with previous knowledge [20,21]. Weights were calculated for each patient as the inverse of the propensity score for the DFS group and the inverse of one minus the propensity score for the conventional group. A standardized difference of 0.10 or less was considered a negligible imbalance between groups. A weighted difference in length of drain stay was estimated using a linear regression model, respectively. *p* values less than 0.05 were considered statistically significant. We used the R v. 4.1.2 (R Foundation for Statistical Computing, Vienna, Austria) for the data analyses.

## 3. Results

A total of 32 patients who underwent the SCIP flap reconstruction were reviewed. Out of them, 14 had the deep fat saving elevation (44%), while 18 had the conventional above-Scarpa elevation (56%). Baseline characteristics are summarized in Table 1.

The flap survived in 14 and 17 patients in the DFS and conventional group, respectively (100 and 94%, *p* = 1.0). Lymphocyst developed in one case of the conventional group and subsided by aspiration. Drain stayed in the donor site for 6 days (IQR: 5.0–6.8 days) in the DFS and eight days (IQR: 7–9 days) in the conventional group, respectively.

### 3.1. Inverse Probability Weighting

After IPW, pseudo-populations were obtained with a size of 31.3 for the conventional group and 35.9 for the DFS group (Table 1). All standardized mean differences were lower than 10%. Table 2 compares postoperative complication rates between the weighted groups. The rates of flap necrosis and donor site morbidities were not significantly different, while the duration of drain stay was longer in the conventional group. The total operative time was similar. In a multiple regression analysis, the performance of the DFS technique decreased the drain stay by 1.6 days (0.4–2.8 days, *p* = 0.01).

### 3.2. Clinical Cases

#### 3.2.1. Case 1

**Figure 2:**
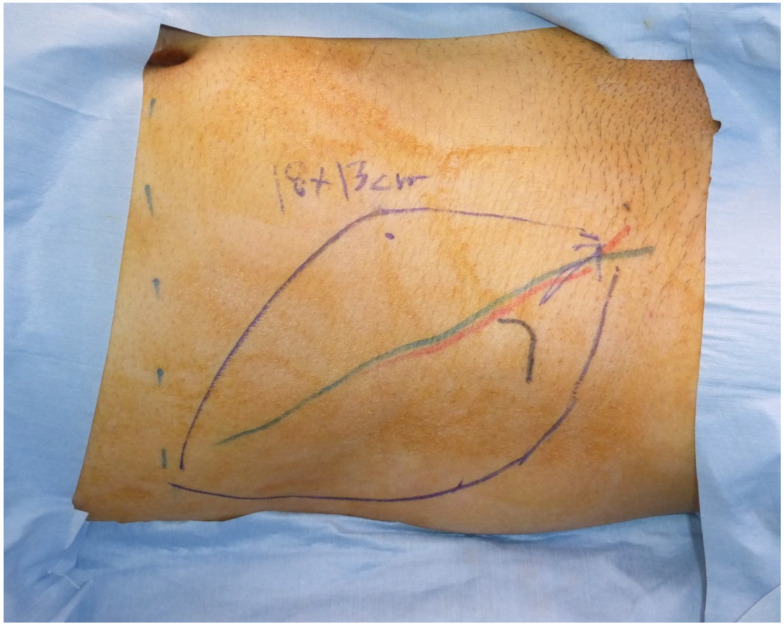
An 18 × 13 cm SCIP flap was designed based on a preoperative marking of the superficial branch of the SCIA.

**Figure 3:**
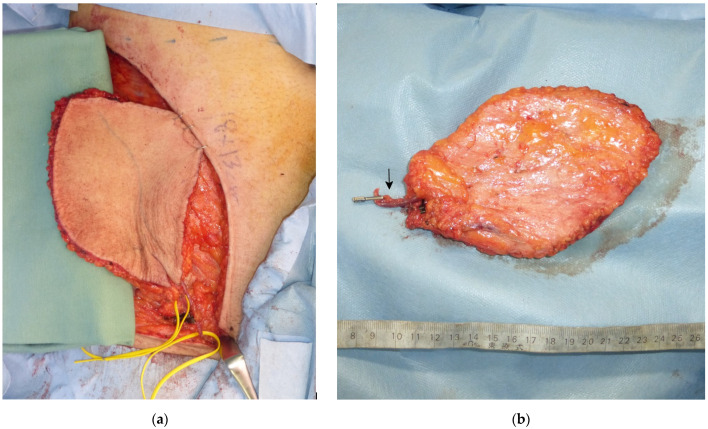
The SCIP flap was elevated saving the deep fat. (**a**) The deep fat remained at the donor site; (**b**) the pedicle of the flap (arrow), the superficial branch of the SCIA, was covered with the fat tissue between the superficial and Camper fasciae in the flap.

**Figure 4:**
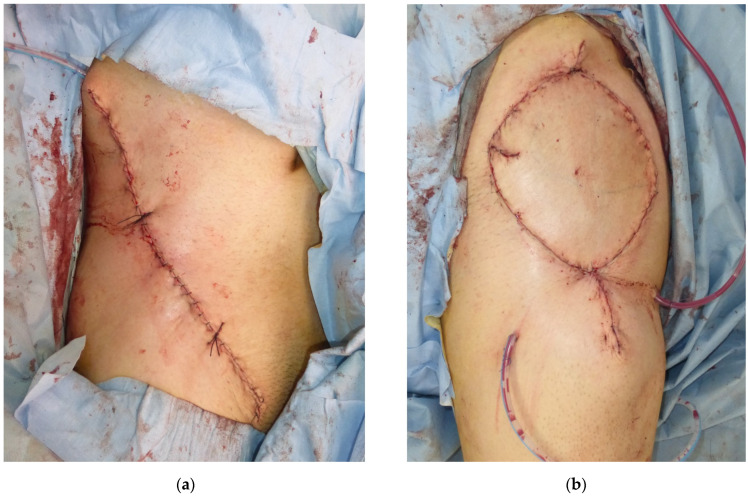
The immediate postoperative views. (**a**) The donor site was closed directly over a suction drain; (**b**) the flap was transferred to the defect.

**Figure 5:**
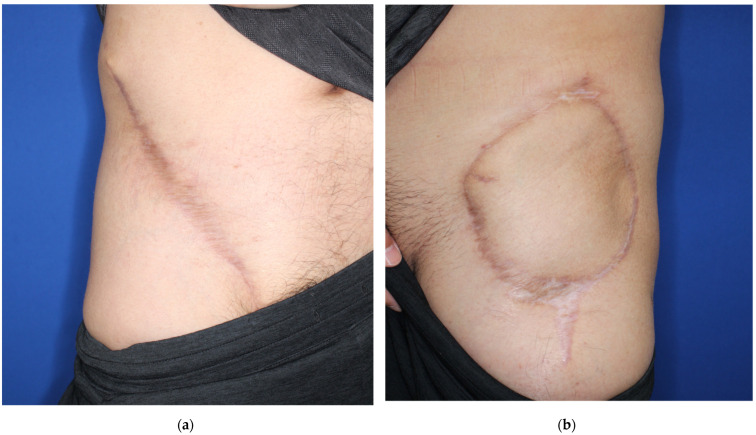
The postoperative photos at a one-year follow-up. (**a**) The donor site healed without complications; (**b**) the flap survived.

A forty-one-year-old man had wide excision of a soft tissue tumor in the left groin. An SCIP flap of 18 × 13 cm based on the superficial branch of the SCIA was harvested in the contralateral groin region (Figure 2). The skin flap was elevated saving the deep soft tissue (Figure 3) and transferred to the defect. The flap survived completely (Figure 4). A suction drain was removed on postoperative day seven. No donor-site morbidity developed (Figure 5).

#### 3.2.2. Case 2

**Figure 6:**
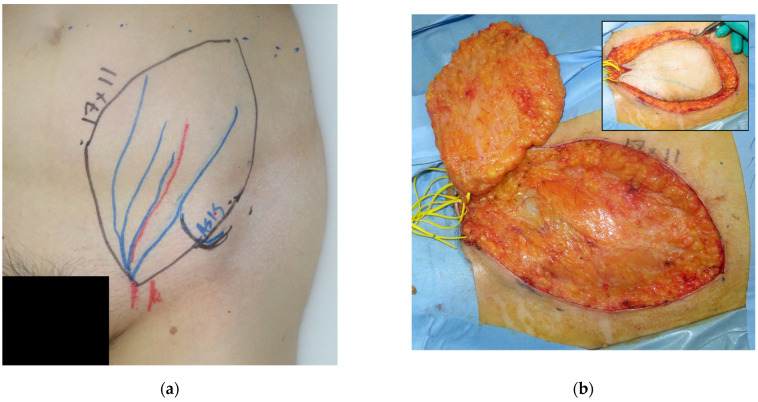

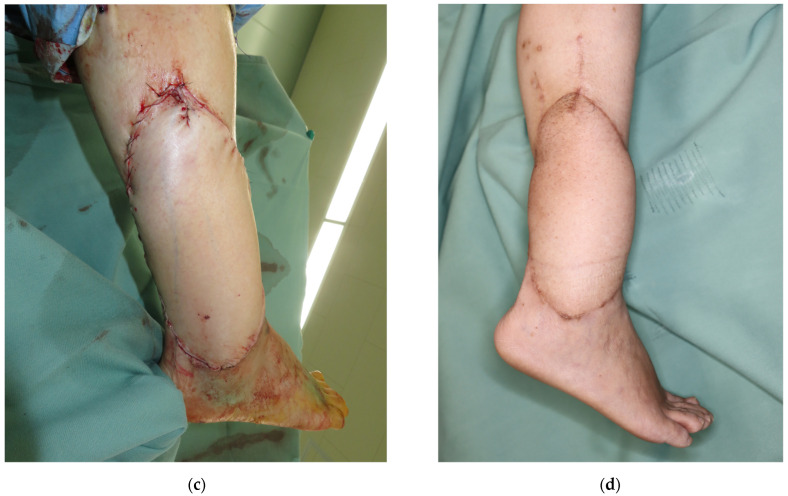
(**a**) Flap design. The flap was elevated based on the SCIA superficial branch. (**b**) The flap was elevated using the DFS technique. (**c**) Immediate postoperative photo. (**d**) Postoperative photo at one year.

A 59-year-old woman was diagnosed with undifferentiated pleomorphic sarcoma in the lower leg. After wide excision, a 15 × 10 cm defect developed. We harvested an SCIP flap of 17 × 11 cm based on the superficial branch using the DFS technique (Figure 6). The flap was transplanted, and the superficial branch and the superficial circumflex iliac vein were anastomosed to the peroneal artery and vein, respectively. The flap and the donor site healed without complications.

## 4. Discussion

We demonstrated a novel elevation technique of the SCIP flap, the Deep Fat Saving technique, in which the deeper fat tissue is saved. Reconstruction results were similar to the conventional SCIP flap elevation. By contrast, the duration of drain stay in the groin donor site was significantly shorter in the DFS group.

An elevation layer of the SCIP flap has been discussed in the pursuit of an ideal reconstruction resource [10,13,14,22]. In this context, the literature has mainly discussed how an SCIP flap is harvested for a thin defect with a skin flap elevated above the superficial fascia or below the dermis. Thin or ultrathin flap transplant can achieve aesthetically favorable reconstruction but requires well-experienced skills. Some surgeons advocated a safer way of thin flap elevation [23].

By contrast, the present study shows a modification of the conventional SCIP flap elevation. An SCIP flap is conventionally elevated above the Scarpa fascia, including the whole layer of the fat tissue. This technique is simple and safe, but prolonged drain stay and lymphatic leakage at the donor site are often problems. In our population, one case of the conventional group developed lymphocyst, and aspiration was necessary. This results from the injury of the lymphatic vessels in the deep fat tissue, and it was alluded that the superficial fascia elevation of the ultrathin flap is less invasive with the lymphatic tissue saved [12]. Considering this speculation, in our novel technique, damage to the lymphatic vessels can be minimized by leaving the deeper fat tissue. However, since the previously reported incidence of lymphorrhea after the SCIP flap elevation was low (<0.5%), the number of patients of the present study was so small that we cannot clearly conclude that the DFS technique offers the same results as the conventional technique.

The “superficial fascia” is the most superficial structure on which the thin flap is elevated [10]. By this fascia, the fat tissue is divided into two layers: superficial and deep. The Camper fascia, which is located above the thin deepest fat [17], is often equated with this superficial fascia. However, based on our accumulated experience, at least two fasciae are distinct in the abdominal and groin fat. Hereby, we advocate an idea that the superficial fascia and the Camper fascia should be considered different structures. By saving the thin fat below the Camper fascia, a relatively thick skin flap is harvested above the Camper fascia. Two patients underwent a revision to correct the bulky flap after the conventional flap elevation. This may be attributed to the patients’ body habitus, not to the elevation layer, since the thin deepest fat hardly affects the thickness of the flap.

**Figure 7:**
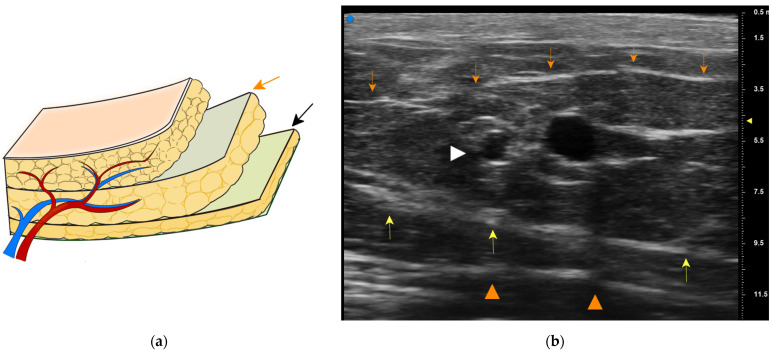
(a) Showing two distinct planes: the superficial fascia (orange arrow) and the Camper fascia (black arrow). (**b**) High-resolution ultrasonographic imaging of the SCIA superficial branch. The superficial branch (white arrow head) is identified between the superficial fascia (orange arrows) and the Camper fascia (yellow arrows). The Scarpa fascia is also distinct (orange arrow heads).

We routinely employ elevation in a proximal-to-distal fashion [9]. A superficial branch of the SCIA is first identified in the proximal region, which is usually between the superficial and the Camper fasciae (Figure 7). The pedicle is rather large and easy to be found with an aid of high-resolution ultrasonography [16]. We then make a circumferential skin incision and dissect toward the Camper fascia. Thus, the pedicle above the Camper fascia is included safely. Furthermore, most of the pedicle is covered by the fat between two fasciae (Figure 3b), contributing to safer transplant with a less possibility of kinking of the pedicle.

The covariates were well balanced through IPW by minimizing the effects of the confounders. Propensity score matching is the most prevailing method to balance the baseline confounders, but this would exclude large proportions of patients. Since the population in the present study is small, the loss of non-matched patients may distort the statistical inference. In the unweighted population, the flap dimension was rather small in the DFS group regardless of a statistically significant difference. However, by IPW, this variable was well-balanced, and confounding was minimized.

### Limitations

The present study is limited by the small number of patients included. The results might be biased due to ethnicity: only Asian patients were enrolled. We did not measure the time of flap elevation. Instead, we employed the total operative time as a surrogate variable. Although we balanced the observed baseline demographics using IPW, unobserved confounders cannot be excluded without a randomized control study.

## 5. Conclusions

The present study demonstrated a novel elevation of the SCIP flap: Deep Fat Saving technique. Inverse probability weighing analysis indicated that drain stay at the donor site was shorter compared to the conventional group, while the rates of flap survival and donor site complications were similar. For patients with indications for the conventional SCIP flap transplant, the DFS technique may be a safe and reliable option.

## Figures and Tables

**Table 1:** Demographics of unweighted population and weighted pseudo-population.

	Unweighted			Weighted		
	Conventional	DFS	*p* Value	Conventional	DFS	SMD ^2^
*n*	18	14		31.3	35.9	
Age >65 years, *n* (%)	8 (44)	7 (50.0)	1.00	12.5 (40)	12.3 (36)	0.076
BMI >25 kg/m2, *n* (%)	3 (17)	3 (21)	1.00	5.7 (18)	6.0 (18)	0.013
Tumor in extremities, *n* (%)	16 (89)	12 (86)	1.00	27.9 (89)	30.2 (89)	<0.01
Flap dimension (median [IQR])	206 [138, 219]	137 [122, 167]	0.05	177.9 [87.1, 216]	143.8 [128, 198]	0.077
DM ^1^, *n* (%)	1 (5.6)	1 (7.1)	1.00	2.2 (7.1)	2.2 (6.6)	0.022
Smoking history *n* (%)	3 (16.7)	3 (21.4)	1.00	6.0 (19)	6.8 (20)	0.023

^1^ DM: Diabetes mellitus, ^2^ SMD: standardized mean difference.

**Table 2:** Surgical results after IPW.

	Conventional	DFS	*p* Value
*n*	31.3	33.9	
Flap survival, *n* (%)	32.0 (95.8)	33.9 (100)	0.32
Partial necrosis, *n* (%)	1.5 (4.8)	0 (0)	0.31
Donor site complications, *n* (%)	1.3 (4.0)	2.4 (7.1)	0.68
Duration of drain stay, days (median (IQR))	8.0 (7.0, 9.0)	6.0 (5.0, 6.7)	<0.01
Operative time, min (median (IQR))	350 (272, 568)	253 (300, 406)	0.85
Flap revision, *n* (%)	0.2 (0.40)	0 (0)	0.127

## Data Availability

The data that support the findings of this study are available from the corresponding author, Y.F., upon reasonable request.
